# Supplementary material for: A hybrid transistor with transcriptionally controlled computation and plasticity
Source: Nat Commun. 2024 Feb 21;15:1598. doi: 10.1038/s41467-024-45759-1 (PMC10881478; doi:10.1038/s41467-024-45759-1)
Supplement: Supplementary file 1 — Supplementary Information [file 41467_2024_45759_MOESM1_ESM.pdf]

Supplementary Information

A Hybrid Transistor with Transcriptionally Controlled Computation and Plasticity

Yang Gao[1], Yuchen Zhou[2,3], Xudong Ji[4,5], Austin J. Graham[1,6], Christopher M. Dundas[1,7], Ismar E. Miniel Mahfoud[1], Bailey M. Tibbett[1], Benjamin Tan[3,8], Gina Partipilo[1], Ananth Dodabalapur[2,3], Jonathan Rivnay[4,5], Benjamin K. Keitz[1*]

[1]McKetta Department of Chemical Engineering, University of Texas at Austin, Austin, TX, 78712, USA
[2]Department of Electrical and Computer Engineering, The University of Texas at Austin, Austin, TX, 78712, USA
[3]Microelectronics Research Center, The University of Texas at Austin, Austin, TX, 78758, USA
[4]Department of Biomedical Engineering, Northwestern University, Evanston, IL, 60208, USA
[5]Simpson Querrey Institute, Northwestern University, Chicago, IL, 60611, USA
[6]Department of Pharmaceutical Chemistry, University of California San Francisco, San Francisco, CA, 94158, USA
[7]Department of Biology, Stanford University, Stanford, CA 94305, USA
[8]Department of Chemistry, University of Texas at Austin, Austin, TX, 78712, USA

*To whom correspondence should be addressed: keitz@utexas.edu

Table of Contents

Methods **3**

Chemicals and Reagents *3*

Bacteria Strains and Culture *3*

Device Fabrication *3*

Device Operation and Electrochemistry *4*

Inoculation Procedure *5*

Spectroscopy *6*

Fluorescence Microscopy *6*

Atomic Force Microscopy *7*

OECT Data Processing *7*

Statistical Methods *8*

Tables **9**

Table S1*. Strains and plasmids used in this study.* *9*

Table S2*. Shewanella Basal Medium (SBM) formulation* *10*

Table S3*. Relevant statistical values.* *10*

Supplementary Figures **11**

Figure S1*. Cell viability and growth in OECTs under varying culture and operation conditions.* *11*

Figure S2*. OECT channel current* I_{DS} *reduction in abiotic and different cell metabolic states.* *12*

Figure S3*. OECT de-doping investigated using electrochemical and spectroscopy methods.* *13*

Figure S4*. OECT responses to* Δ*mtrC strains carrying NAND and NOR Boolean gate plasmids.* *14*

Figure S5. *Modulation of the OECT synaptic behavior with varying pulse conditions and strains.* *15*

Figure S6*. Cartoon illustration of the major OECT channel fabrication and assembly steps.* *16*

Figure S7*. Morphological characterization of PEDOT:PSS channel and gate-tip coating.* *17*

Figure S8*. OECT response to different extracellular electron transfer (EET) mechanisms.* *18*

Figure S9*. Channel current* I_{DS}/I_{DS0} *curves of strains carrying the Boolean logic gates plasmids.* *19*

Figure S10*. Growth curves of mutant strains carrying the Boolean logic gates plasmids.* *19*

Figure S11*. Measured channel currents* I_{DS} *and electrode potentials responding to gate pulses.* *20*

Figure S12*. Paired-pulse responses of abiotic OECTs at varying electrochemical doping states.* *20*

Figure S13*. Electrochemical response of the hybrid OECTs under varying oxygen conditions.* *21*

References **21**

Methods

Chemicals and Reagents

PEDOT:PSS aqueous suspension (Clevios™ PH1000, Heraeus Epurio LLC), ethylene glycol (anhydrous 99.8%, Sigma-Aldrich), sulfuric acid (H_2SO_4, 95.0-98.0 %, Sigma-Aldrich), hydrogen peroxide (H_2O_2, 30 wt% in water, Sigma-Aldrich), sodium DL-lactate ($NaC_3H_5O_3$, 60% in water, TCI), sodium fumarate ($Na_2C_4H_2O_4$, 98%, VWR), HEPES buffer solution ($C_8H_{18}N_2O_4S$, 1 M in water, pH = 7.3, VWR), potassium phosphate dibasic (K_2HPO_4, Sigma-Aldrich), potassium phosphate monobasic (KH_2PO_4, Sigma-Aldrich), sodium chloride (NaCl, VWR), ammonium sulfate ($(NH4)_2SO_4$, Fisher Scientific), magnesium(II) sulfate heptahydrate ($MgSO_4 \cdot 7H_2O$, VWR), Wolfe's Trace Mineral Mix (ATCC), casamino acids (VWR), isopropyl ß-D-1-thiogalactopyranoside (IPTG, Teknova), anhydrotetracycline hydrochloride (aTc, Sigma-Aldrich), 3-oxohexanoyl-homoserine lactone (OC6, Sigma-Aldrich), kanamycin sulfate ($C_{18}H_{38}N_4O_{15}S$, Growcells), Riboflavin 5′ -monophosphate sodium salt hydrate (>93 %, TCI), and LIVE/DEAD® BacLight™ Stain (L7012, Invitrogen), were used as received. Two-part silicone elastomer (Sylgard™ 184, Electron Microscopy Sciences) was used according to manufacturer instructions. All media components were autoclaved or sterilized using 0.22 μm PES filters.

Bacteria Strains and Culture

Bacterial strains and plasmids are listed in Table S1. Cell cultures were prepared from bacterial stocks stored in 20% glycerol at −80 °C. The stocks were streaked onto agar plates containing LB (for wild-type and knockout strains) or LB with 25 μg/mL kanamycin (for plasmid-harboring strains), and subsequently grown overnight at 30 °C for *Shewanella* and 37 °C for *E. coli*. Single colonies from the plates were picked and inoculated into Shewanella Basal Medium (SBM, Table S2) amended with 0.05% trace mineral supplement, 0.05% casamino acids, and supplemented 20 mM sodium lactate (2.85 μL of 60% w/w sodium lactate per 1 mL culture) for *Shewanella* and 20 mM glucose (10 μL of 2 M glucose per 1 mL culture) for *E. coli* as the electron donor, unless otherwise noted. Aerobic cultures were pregrown in 15 mL culture tubes at 30 °C and 250 rpm shaking. Anaerobic cultures were pregrown using the same procedure outlined above, but with argon purged growth medium in a nitrogen-filled glovebox (S1200, Vigor) and supplemented with 40 mM sodium fumarate (40 μL of 1 M sodium fumarate per 1 mL culture) as the electron acceptor. Additional 25 μg/mL kanamycin (10 μL of 2.5 mg/mL kanamycin per 1 mL culture) was supplemented to the pregrowth medium of plasmid-harboring strains. Aerobically pregrown cultures were washed 3x using the sterile SBM growth medium and adjusted to an OD_{600} of 1-3.5 (NanoDrop 2000C) before being transferred into the glovebox. For steady-state protein expression, strains were pregrown anaerobically without inducer(s) for 6 h before being diluted 1:25 into inducer-containing media (from 1000x stocks) and grown for 18-24 hours.

Device Fabrication

Three versions of the OECTs were used: small channel OECTs, 2-electrode versions of the small channel OECT without the gate, and a large channel OECT. The small channel OECTs were used for the majority of experiments with the following exceptions. In direct channel reduction experiments (Figure 2h, Figure S3g), the 2-electrode versions of the small channel OECT were used and noted as 2-electrode devices or no gate. In UV-vis spectroscopy (Figure 2g, Figure S3c – S3e), the large channel OECTs were used to fit the laser aperture of the instrument. The large channel OECTs were labeled as 'large channel OECTs' and the small channel OECTs were noted as original three-terminal OECTs or without any specific naming. OECTs were fabricated according to prior work[1]. Quartz microscopic slides (FQ-S-003, AdValue Technology) were cleaned with soapy water, acetone, and isopropyl alcohol, and dried with nitrogen before oxygen reactive-ion etching (RIE, 150 W, 50 sccm, 120 s). Quartz slides were coated with a photoresist layer (AZ5209E) and lithographically patterned to define the electrode layout. Subsequently, Au electrodes (100 nm) with a Ti adhesion layer (10 nm) were thermally evaporated on the quartz slides, and excess materials were removed with acetone lift-off. Another photolithographic pattern was formed to define the PEDOT:PSS region over the channel and the tip of the gate to obtain a width/length of 150 μm x 10 μm and 500 μm x 500 μm, respectively. The PEDOT:PSS (Clevios^{TM} PH1000) was filtered (0.22 μm PES filters) and spun cast on the pattered quartz slides, followed by hot plate drying at 90 °C for 15 min and acetone lift-off. To increase the conductivity, PEDOT:PSS films were immersed in ethylene glycol at 90 °C for 3 min over a hot plate. The as-fabricated PEDOT:PSS film had an average thickness of 26.5 nm for the channel and 38.3 nm for the gate-tip layer. Polydimethylsiloxane (PDMS, Sylgard^{TM} 184) with 9 % wt curing agent was drop cast and cured for 48 hours at room temperature to ensure surface smoothness. The OECT chambers and fluid access ports in the PDMS sheets were manually cut with a hole punch.

The OECTs with the larger channel size were only used for UV-vis measurement. The electrodes were fabricated in the same way as the smaller OECT. The PEDOT:PSS channel was fabricated by drop-casing 80 μL PEDOT:PSS over the entire slide, followed by 20 minutes of air-drying and heating at 90°C for 20 minutes. Afterward, the excessive PEDOT:PSS film was removed with cotton swabs soaked with 70% ethanol. Conductivity enhancement was achieved by EG treatment of 10 min at 90 °C. Silicon spacers (0.5 mm, GBL664581, Sigma-Aldrich) were hand cut to create the OECT chambers and only used with the large channel OECTs.

Device Operation and Electrochemistry

Shewanella Basal Medium (SBM) amended with 0.05% casamino acids and 1X Wolfe's Trace Mineral Mix was used as the base electrolyte except for the carbon source comparison experiments (Figure 2e, Figure S2d) where the SBM was only amended with 1X Wolfe's Trace Mineral Mix. When noted, the SBM was further supplemented with 40 mM sodium fumarate to support cell growth. Before each experiment, the OECT slides and PDMS sheets were autoclaved separately and assembled in the biosafety cabinet. OECT experiments were conducted in a nitrogen-filled glovebox to create the anaerobic condition except for UV-vis spectroscopy which was conducted under ambient conditions. Media and solutions stocks were purged with argon for 15 min and stored in the glovebox. A multichannel potentiostat (MultiPalmSens4, PalmSens BV) was used for the electrochemical measurements. During continuous OECT operation, unless

otherwise noted, the gate (V_{GS}) and drain (V_{DS}) voltages were biased at 0.2V and -0.05 V, respectively. Transfer curves were measured with a gate scan rate of 20 mV/s, except in the artificial synaptic hysteresis measurement where a rate of 10 mV/s was used. For all the experiments, OECTs were stabilized in the glovebox with abiotic electrolytes and constant bias voltages for 72 hours before inoculation or measurements.

In the electrode potential measurements with Ag/AgCl pellet reference electrode (550010, A-M Systems), the Ag/AgCl electrodes were directly inserted into the OECT chamber without salt bridges. The same Ag/AgCl pellet electrodes were used as the gate for the large channel OECTs in abiotic UV-vis measurements. Hybrid OECT experiments with electroactive bacteria were conducted between 18 - 36 hours after inoculation (initial OD_{600} at 0.01). Synaptic measurements were conducted after I_{DS} was stabilized for at least 1 minute at V_{GS} = 0 V.

Inoculation Procedure

For aerobic culture growth, SBM supplemented with 20 mM lactate was used for cell growth. Following aerobic overnight growth, the cultures were triple-washed by centrifugation using the same growth medium to remove byproducts. The optical density of the washed cultures was measured before transferring them into the glovebox. side the glovebox, the cell cultures were diluted to create an intermediate inoculum with a cell density 10-fold higher than the target OD for final inoculation. This dilution used the same electrolyte solution that filled the OECT. Finally, the OECTs were inoculated using a 1:9 volume ratio of the intermediate cell culture to the OECT electrolyte. For instance, *S. oneidensis* supplemented with 1 μM exogenous FMN with inoculum OD_{600} of 0.05 was prepared from aerobically grown cell cultures. The triple-washed cell cultures (average OD_{600} at 3.38) were brought into the glovebox and diluted to the intended OD_{600} of 0.5 with SBM supplemented with 1 μM exogenous FMN, creating the intermediate cultures. Then 5 μL of these intermediate cultures were inoculated into the OECTs containing 45 μL of SBM supplemented with 1 μM FMN, achieving a final inoculation OD_{600} at 0.05.

For steady-state expression conditions, cells were anaerobically cultured in SBM supplemented with 20 mM lactate and 40 mM fumarate, along with specific inducer(s). To maintain consistent induction strength and to prevent cell growth from affecting OECT readouts, electrolyte in the OECTs were SBM supplemented with 20 mM lactate and specific inducer(s), but lacked fumarate. Prior to induction, strains were grown statically and anaerobically without inducer(s) for 6 hours. Subsequently, cell cultures were diluted 1:25 into media containing inducers (prepared from 1000x concentrated stocks) and incubated for an additional 18-24 hours. After this induction period, the cell cultures were directly inoculated into the OECTs at a 100-fold overall dilution without prior washing. Specifically, cells were first diluted 10-fold with the fumarate-free OECT electrolyte forming the intermediate cultures. The final inoculation of the OECTs was achieved by adding the intermediate cell culture to the OECT electrolyte at a 1:9 volume ratio.

In the carbon source experiment, SBM amended with 1X Wolfe's Trace Mineral Mix was used as the base electrolyte. Aerobically grown *S. oneidensis* MR-1 cells culture were triple-washed with SBM without a carbon source, then the cell cultures were kept at room temperature for 3 hours to

induce starvation conditions[2]. Afterward, cell cultures were washed again and their OD_{600} was measured. Subsequently, cell cultures were brought into the glovebox and diluted to obtain the intermediate stocks with an intended OD_{600} of 0.1. The dilutions were performed with SBM supplemented with either 20 mM lactate, 20 mM pyruvate, 20 mM acetate, or no carbon source. Finally, the intermediate stocks were inoculated into OECTs containing the SBM supplemented with the respective carbon source or no carbon source at a ratio of 1:9, achieving a final inoculation OD_{600} of 0.01.

In cell viability experiments, aerobically grown *S. oneidensis* MR-1 cell cultures were triple-washed with SBM supplemented with 20 mM lactate. For *E. coli* cultures, the lactate was replaced with 20 mM glucose. The densities for the washed cell cultures were measured by OD_{600} and the *S. oneidensis* cultures were allocated to 3 parts: live, heat-killed, and lysed cells. Heat-killed cells were obtained by incubating at 80 °C for 20 min. Lysed cells were obtained by sonication (Qsonica 55, Qsonica LLC) for 90 s at 4 °C. The output power was set to 15 W with "ON" and "OFF" intervals of 10 s and 5 s, respectively. Then cell cultures were brought into the glovebox and diluted with SBM supplemented with 20 mM lactate to obtain the intermediate cultures at an intended OD600 of 0.1. The intermediate cultures were inoculated into the OECTs at a ratio of 1:9, achieving a final inoculation OD_{600} of 0.01. The *S. oneidensis* supernatants were obtained by filtering (0.22 µm PES filters) the cell cultures (initial OD600 at 0.01) anaerobically grown in the glovebox with SBM supplemented with 20 mM lactate. The entire volume of OECT electrolyte was replaced with the supernatant during inoculation.

Spectroscopy

The UV-Vis spectroscopy of the PEDOT:PSS channel was measured from 190 nm to 1100 nm (Agilent 8453 UV-Visible Spectroscopy System). A custom sample holder was 3D printed to fit the OECT slides to the instrument. Measurements were blanked with devices lacking the PEDOT:PSS channel. When bacteria cells were present in the sample, the blank devices were likewise inoculated with the same inoculum.

Fluorescence Microscopy

Microscopy was performed using a Nikon Ti2 Eclipse inverted epifluorescence microscope. Immediately after the 24-hour operation in the glovebox, OECTs were gently washed 2x by refreshing the electrolyte with the sterile SBM supplemented with 0.05% trace mineral supplement, 0.05% casamino acids. Then, the PDMS sheets were replaced with a 0.5 mm silicon spacer to ensure the sample thickness was compatible with the working distance of the microscope. Subsequently, the OECTs were gently washed with SBM containing 0.05% trace mineral supplement, 0.05% casamino acids, and LIVE/DEAD® BacLight™ Stain mix (3 µL of SYTO 9 and propidium stocks per 1 mL) at a final solution volume of 10 µL per OECT chamber. The OECTs were then sealed with coverslips, covered with aluminum foil, and transferred out of the glovebox for microscope imaging. Bacterial counts were performed using ImageJ software.

Atomic Force Microscopy

The AFM scans were conducted using a DriveAFM (Nanosurf AG). The cantilevers (Dyn190Al) were driven with the photothermal laser (CleanDrive) under dynamic mode. OECTs were randomly selected from two fabrication batches: 3 slides of the as-fabricated OECTs (8 OECTs per slide) were used as pre-inoculation samples, and 3 cleaned OECT slides post the 48-hour inoculation served as post-inoculation samples. Two OECTs per slide were randomly chosen for AFM scans using Nanosurf CX software for data acquisition. Images for PEDOT:PSS film thickness were acquired at 90 µm x 90 µm and processed with Nanosurf CX software to correct background variations. Topology and phase images were acquired at 500 nm x 500 nm and analyzed without further process. Grain sizes were fitted by the Watershed method with the resulting histograms generated using Gwyddion software.

OECT Data Processing

Measured OECT data were processed using GraphPad Prism9 and MATLAB (R2021b update 1). The measured I_{DS} data were normalized to the initial value before inoculation (I_{DS0}) before fitting. The I_{DS} decay rate constants for all samples except the lysed *S. oneidensis* were obtained by fitting I_{DS}/I_{DS0} data with an exponential decay model:

$$i_{DS}(t) = e^{k*t} \tag{1}$$

Where t is time in hours, k is the fitted I_{DS} decay rate constant. Fitting was performed with the built-in one phase decay function in GraphPad Prism9. The I_{DS}/I_{DS0} data for lysed *S. oneidensis* samples were fitted to a simple linear regression model:

$$i_{DS}(t) = I_{DS0} + \beta t \tag{2}$$

Where t is time in hours, the slope β is used as the rate of change. Fitting was performed with the built-in simple linear regression function in GraphPad Prism9.
The response function of FMN concentrations was modeled with a four-parameter logistic regression function in terms of I_{DS} decay rate constants (noted here as r):

$$\frac{r}{R_{max}} = \frac{[S]^n}{\left(K_{\frac{1}{2}}\right)^n + [S]^n} \tag{3}$$

Where n is the Hill coefficient, $[S]$ is the FMN concentrations, R_{max} is the maximum rate constant, $K_{\frac{1}{2}}$ is the half-maximum concentration constant. Fitting was performed with the built-in four-parameter dose-response function in GraphPad Prism9.
In synaptic experiments, MATLAB scripts were used to process the measured data. The 'Vgs_pulse_pair.m' script was used to identify and extract the channel current I_{DS} peak values using islocalmax function (Figure 5d, 5e, 5g - 5i, Extended Data Figure 5a - 5e). The 'IDS_VGS_Pul_long.m' script implemented median filtering to remove pulses and extract the baseline for channel current I_{DS} responding to continuous gate pulse inputs. The filtering was

achieved by using the medfilt1 function (Figure 5f and Figure S5f). Extracted I_{DS} curves were fitted to an exponential model b(1)*exp(b(2)*x(:,1))+b(3) with fitnlm function to obtain the rate constants, where b(1), b(2), and b(3) are fitting coefficients, x is time in seconds.

All scripts are available through the Texas Data Repository.

Statistical Methods

Independent samples t-tests were conducted using the built-in t-test function with the unpaired option in GraphPad Prism9.

In the 2-input Boolean gate figures (Figure 4c and 4d, Figure 5h and 5i), statistical tests were performed using R (version 4.2.3) with multicomp package (version 1.4-23). In brief, we employed a linear model fitting approach to determine the interaction term between the two inputs noted as Factor1 and Factor2, specifically lm(Results ~ Factor1 * Factor2 – 1). Then, based on the significance of the interaction terms, different linear contrasts were tested for general linear hypotheses, specifically glht(model, linfc = contrast). The significance of the interaction term was examined for $p < 0.05$. If the interaction term was significant, the following linear contrasts were evaluated for each specific logic gate and model parameterization:

$$\text{NAND gate: } H_0\text{: } \mu_{++} + \frac{-1}{3}(\mu_{--} + \mu_{+-} + \mu_{-+}) = 0 \quad (4)$$

$$\text{NOR gate: } H_0\text{: } \frac{-1}{3}(\mu_{++} + \mu_{+-} + \mu_{-+}) + \mu_{--} = 0 \quad (5)$$

The contrast matrices for the NAND and NOR logic gates in the general linear hypothesis test are as follows: $(^2/_3, -^2/_3, -^2/_3, -^1/_3)$ for NAND gate, and $(-^2/_3, ^2/_3, ^2/_3, 1)$ for NOR gate. These contrast matrices were calculated by algebraic substitution given the following coefficient/parameter relationships defined by our parameterization of the linear model, specifically: μ_{++} = coef[1], μ_{+-}= coef[1] + coef[3], μ_{-+}= coef[2], μ_{--}= coef[2] + coef[3] + coef[4]. The resulting p-values and the interaction term are outlined in Table S3.

If the interaction term was not significant, a linear model without interaction was fit to the data, and the model was tested with new contrast matrix as: $(1, -^1/_3, -^1/_3, -^1/_3)$ for the NAND gate, and $(-^1/_3, 1, -^1/_3, -^1/_3)$ for the NOR gate. The p-values are outlined in Table S3 without an interaction term.

Tables

Table S1. Strains and plasmids used in this study.

Strain and Plasmid	Description	Source
**Strains**		
Escherichia coli		
MG1655	Wild-type strain	Lydia Contreras, University of Texas at Austin
Shewanella oneidensis		
MR-1	MR-1 (ATCC700550) wild type strain	American-Type Culture Collection
△*mtrC*	JG596, deletion of genes *mtrC*, *omcA*, and *mtrF*.	Jeffrey Gralnick, University of Minnesota[3]
△Mtr	JG1194, deletion of genes *mtrC*, *omcA*, *mtrF*, *mtrA*, *mtrD*, *dmsE*, *SO4360*, *cctA,* and *recA*.	Jeffrey Gralnick, University of Minnesota[4]
△*bfe*	deletion of genes *SO0702.*	Jeffrey Gralnick, University of Minnesota[5]
△*lysis*	S2933, deletion of genes *SO2966* and SO2974.	Lydia Contreras, University of Texas at Austin[6]
**Plasmids**		
pCD8	Empty Buffer gate	Ref.[7]
pCD24r1	*mtrC* Buffer gate (sRBS1$_{mtrC}$)	Ref.[7]
pAT4	*Mtr* Buffer gate	This work
pNAND-*mtrC*	*mtrC* NAND Boolean logic gate	Ref.[8]
pNAND-*sfgfp*	*sfgfp* NAND Boolean logic gate	Ref.[8]
pNOR-*mtrC*	*mtrC* NOR Boolean logic gate	Ref.[8]
pNOR-*sfgfp*	*sfgfp* NOR Boolean logic gate	Ref.[8]

Table S2. Shewanella Basal Medium (SBM) formulation

Ingredient	Quantity per 1 L
K_2HPO_4	225 mg
KH_2PO_4	225 mg
NaCl	460 mg
$(NH_4)_2SO_4$	225 mg
$MgSO_4{*}7H_2O$	117 mg
HEPES	100 mL of 1 M HEPES
Casamino acids	5 mL of 10% casamino acids in ddH_2O, if needed
Wolfe's Mineral Mix	5 mL of Wolfe's Mineral Mix, if needed
ddH_2O	Adjust volume to 1 L and pH to 7.2

Table S3. Relevant statistical values

Data Sets	pNAND	pNOR
**Source Potential (V_S)**	2-way ANOVA interaction $p= 0.00313$<br><br>Contrast test $p = 0.00204$	2-way ANOVA interaction $p= 1.84 \times 10^{-5}$<br><br>Contrast test $p = 2.36 \times 10^{-7}$
**Synaptic Conductance Change ($\triangle G$)**	1-way ANOVA contrast test $p = 0.00306$	1-way ANOVA contrast test $p = 0.00152$

Supplementary Figures

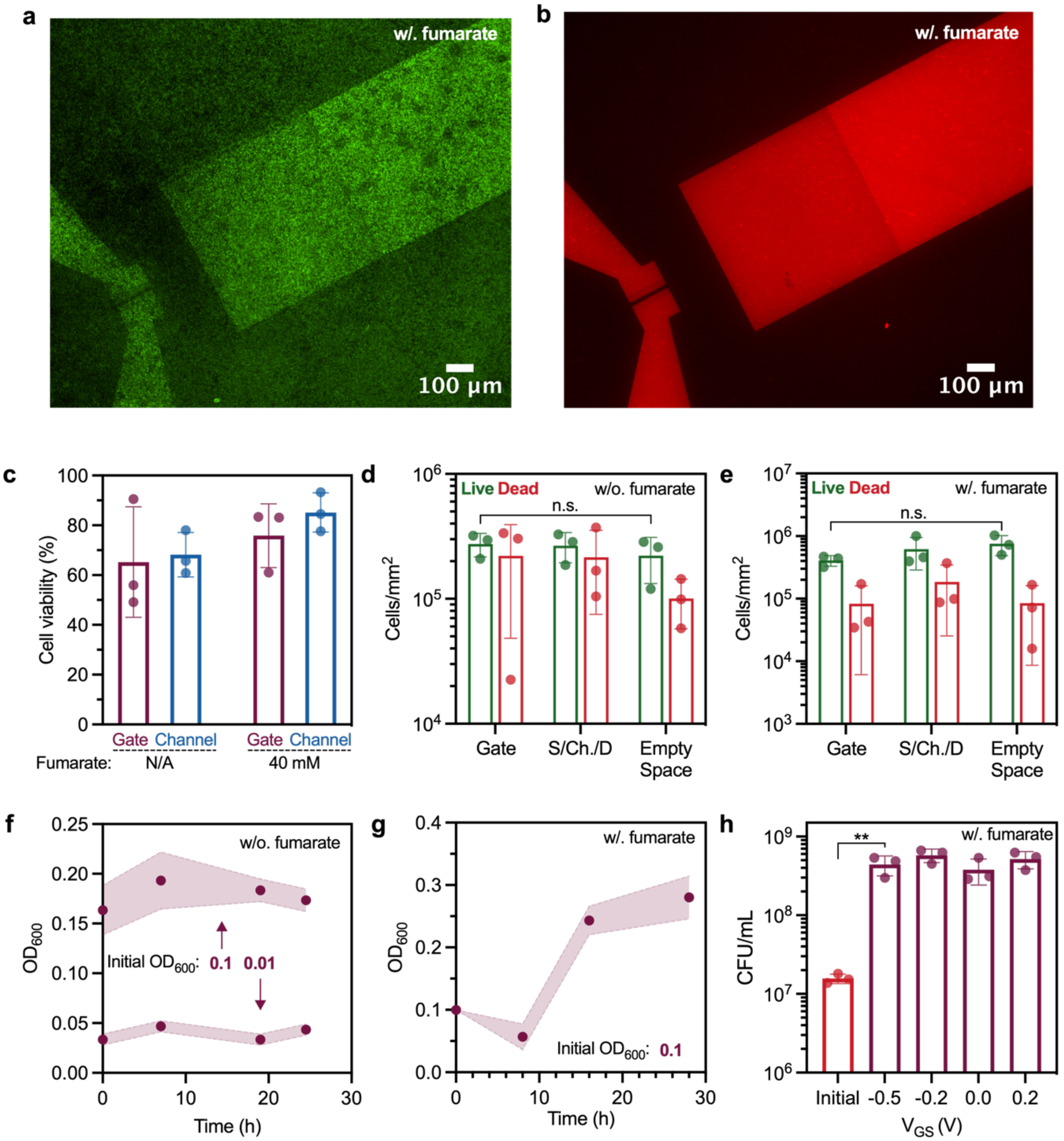

Figure S1. Cell viability and growth in OECTs under varying culture and operation conditions. Representative fluorescent microscopy images showing OECTs operated with constant drain voltage V_{DS} = -0.05V and gate voltage V_{GS} = 0.2V. Cells were supplemented with 20 mM lactate and 40 mM fumarate. LIVE/DEAD® BacLight™ cell assay showing (**a**) live cells in green and (**b**) dead cells in red. (**c**) Cell viability derived from the fluorescent microscopy images. (**d, e**) Cell counts over OECT gates, source/channel/drain region (denoted as S/Ch./D), and spaces elsewhere (denoted as empty spaces) for cells supplemented with 20 mM lactate, and (**d**) without or (**e**) with 40 mM fumarate. (**f, g**) Optical Density at 600 nm (OD_{600}) was measured from

OECTs biased at V_{DS} = -0.05V and V_{GS} = 0.2V, cells supplemented with 20 mM lactate, and (**f**) without or (**g**) with 40 mM fumarate. The shaded region indicates the range of standard deviation. (**h**) Colony forming units (CFUs) were counted 24 hours after OECTs operation with constant V_{DS} = -0.05 V and V_{GS} biased at -0.5 V, -0.2 V, 0.0 V, or 0.2 V. CFU/mL p value for the indicated pair p = 0.0042. Cells were supplemented with 20 mM lactate and 40 mM fumarate. Data show the mean ± SD of 3 biological replicates, unpaired two-tailed Student's t-tests were performed without adjustments for multiple comparisons, n.s. represents $p > 0.05$.

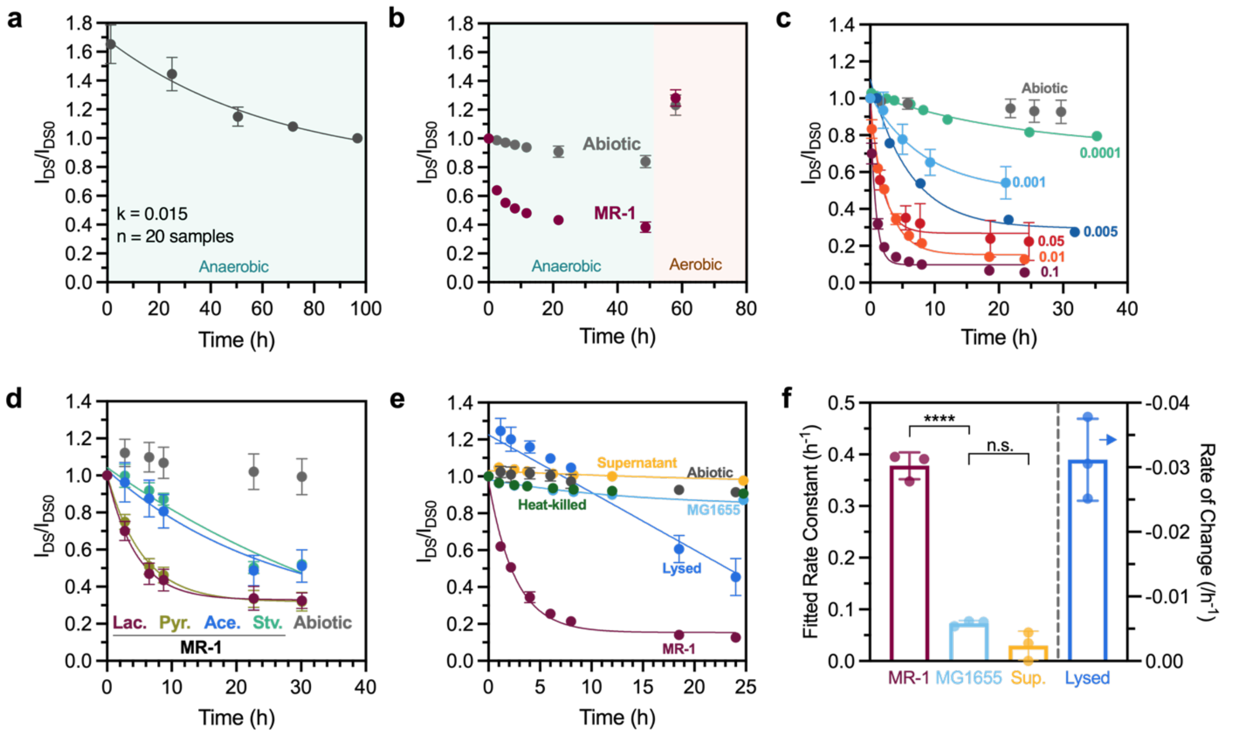

Figure S2. OECT channel current I_{DS} reduction in abiotic and different cell metabolic states. The I_{DS}/I_{DS0} curves during (**a**) anaerobic stabilization without bacteria cells and (**b**) 48-hour operation followed by exposure to the oxygen in the ambient environment. The I_{DS}/I_{DS0} curves of *S. oneidensis* MR-1 inoculated OECTs with (**c**) varying inoculation OD_{600} numbers or (**d**) supplemented with either 20 mM sodium lactate (Lac.), 20 mM sodium pyruvate (Pyr.), or 20 mM sodium acetate (Ace.) as the electron donors. No carbon source was supplied to the starved cells (Stv.) (**e**) The I_{DS}/I_{DS0} curves for living, lysed, and heat-killed *S. oneidensis* MR-1 cells, as well as living *E. coli* MG1655 cells. (**f**) The I_{DS} decay rate constants for living *S. oneidensis* and *E. coli* cells, as well as *S. oneidensis* supernatant. Fitted rate constant p values for the indicated pair p = 3.8 × 10^{-5}. The lysed *S. oneidensis* curves were fitted using a linear regression model. Cells were supplemented with 20 mM lactate for *S. oneidensis* MR-1 and 20 mM glucose for *E. coli* MG1655, no electron acceptor was added. Initial inocula were adjusted to OD_{600} of 0.01. Data show the mean ± SD of 3 biological replicates, unpaired two-tailed Student's t-tests were performed without adjustments for multiple comparisons, n.s. represents $p > 0.05$.

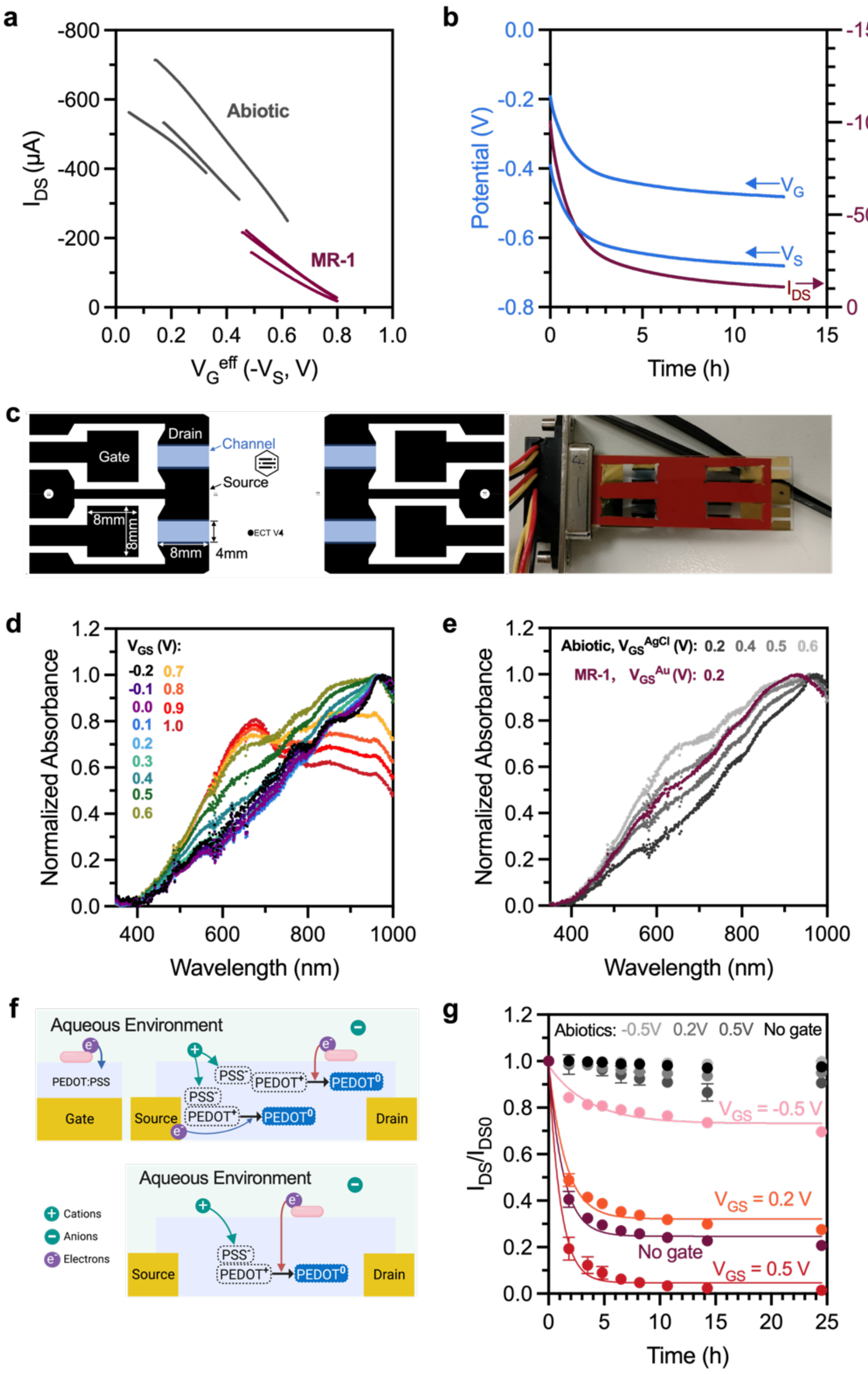

Figure S3. OECT de-doping investigated using electrochemical and spectroscopy methods. (**a**) Transfer curves plotted using effective gate voltage (V_G^{eff}) values with respect to Ag/AgCl pellet reference electrodes. (**b**) Gate (V_G)and source (V_S) potentials with respect to the Ag/AgCl pellet reference electrode plotted with the channel current I_{DS} for an OECT inoculated with *S. oneidensis* MR-1. (**c**, left) Schematic and (**c**, right) photo-image of the large channel OECT used exclusively for the UV-Vis instrument. UV-Vis spectra were collected for (**d**) abiotic PEDOT:PSS channel under different Ag/AgCl pellet gate bias voltages, and (**e**) for *S. oneidensis* MR-1 inoculated channel overlayed with abiotic channels. (**f**) Cartoon illustrations comparing the

cellular extracellular electron transfer (EET) de-doping process of OECT and the 2-electrode device. (**g**) The channel current I_{DS}/I_{DS0} curves of the 2-electrode devices and OECTs under constant gate bias conditions. *S. oneidensis* MR-1 was used for inoculum with an inoculation OD_{600} of 0.01. Data in panel (g) show the mean ± SD of 3 biological replicates. Figure (f) created with BioRender.com.

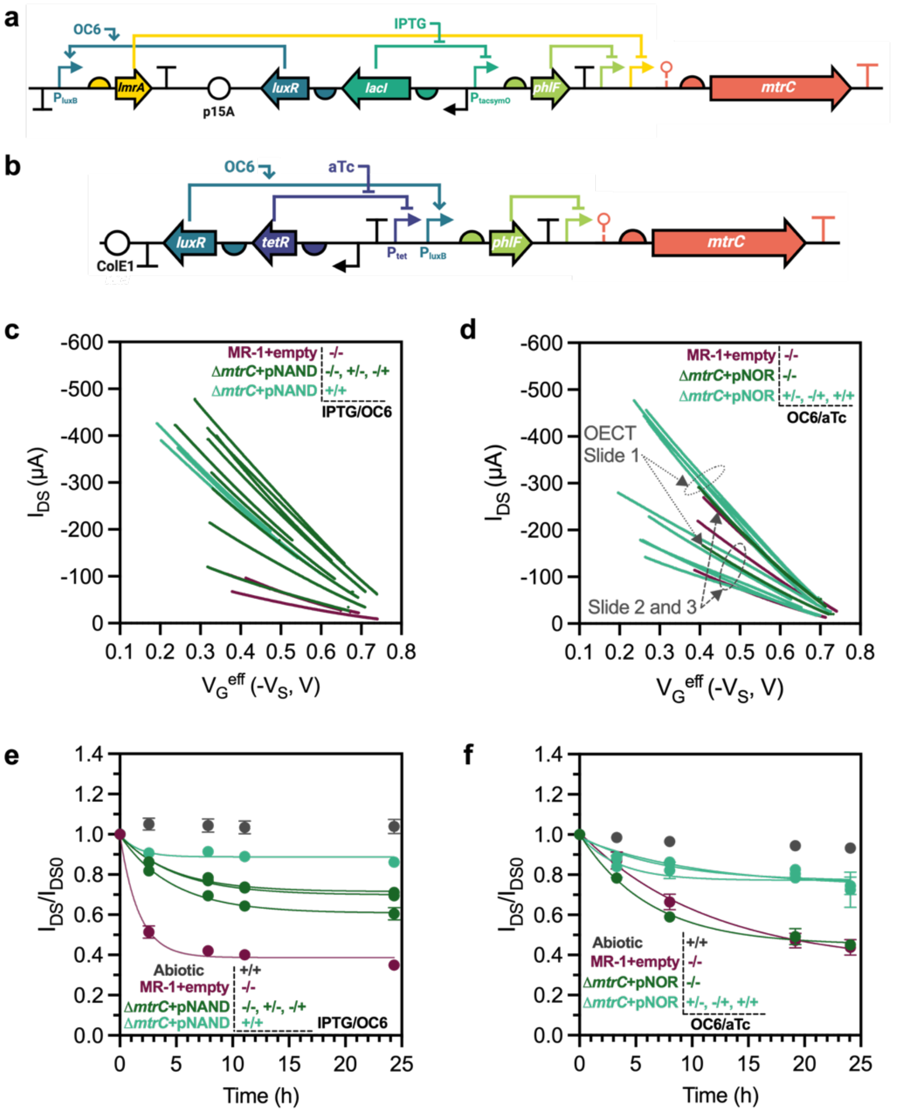

Figure S4. OECT responses to Δ*mtrC* strains carrying NAND and NOR Boolean gate plasmids. Cartoon illustrations of the plasmid architecture of the (**a**) NAND and (**b**) NOR Boolean gates expressing *mtrC*. The Δ*mtrC* mutants carrying the corresponding Boolean gate plasmids were brought to steady-state MtrC expression with combinations of 500 μM IPTG, 200 nM OC6, and 10 nM aTc inducers. Transfer curves of the induced (**c**) NAND and (**d**) NOR gates samples. The channel current I_{DS}/I_{DS0} curves for (**e**) NAND and (**f**) NOR gates samples with different inducer combinations. Data in panels (e) and (f) show the mean ± SD of 3 biological replicates. Figure (a) and (b) created with BioRender.com.

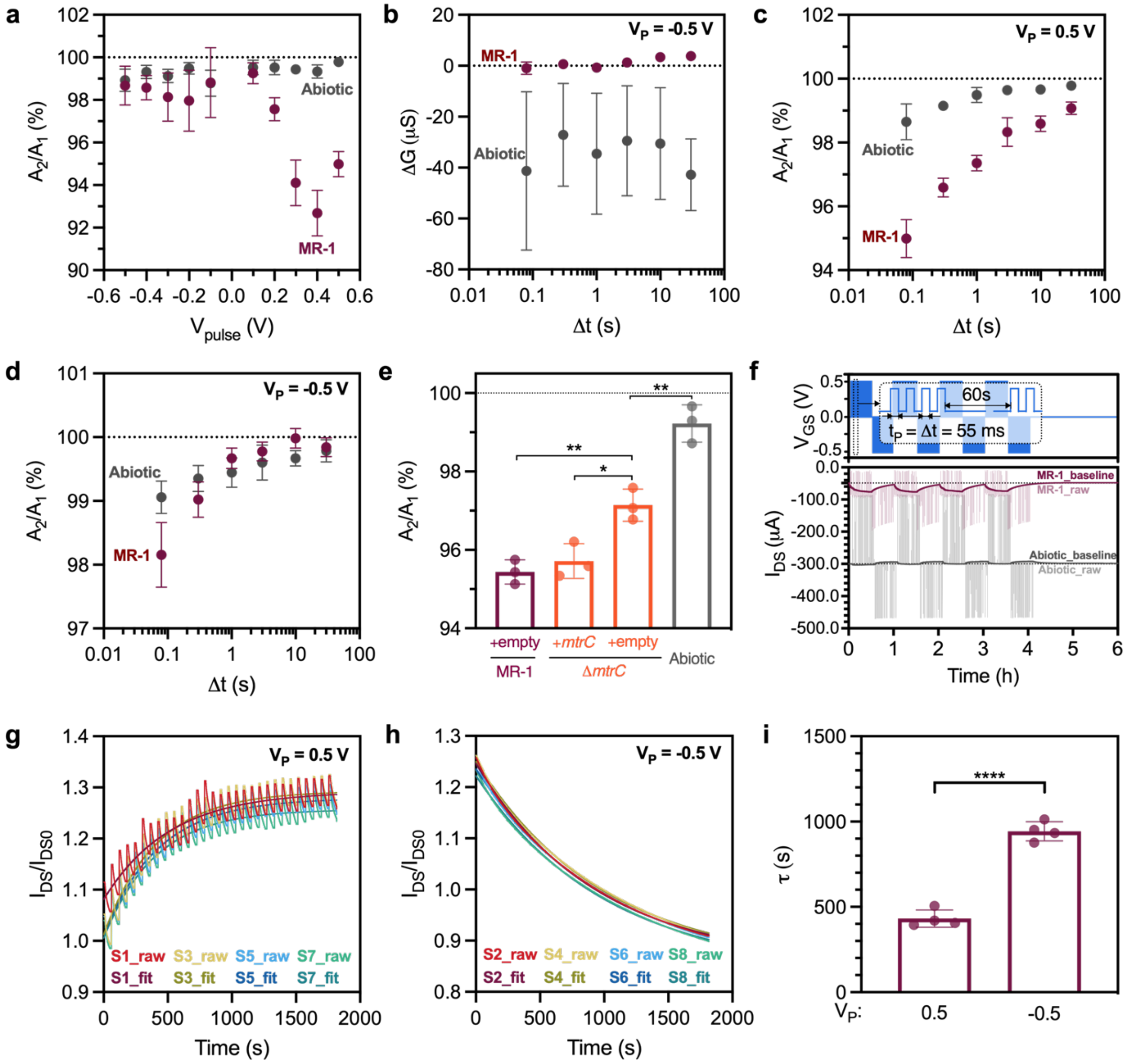

Figure S5. Modulation of the OECT synaptic behavior with varying pulse conditions and strains. (**a**) A2/A1 index plotted with varying pules voltage V_P, while pules duration t_P and pules interval Δt were fixed at 80 ms. (**b**) OECT channel conductance changes ΔG with varying pulse interval Δt, fixed t_P = 80 ms and V_P = -0.5 V. A2/A1 index for OECTs with varying pulse interval Δt, fixed t_P = 80 ms and V_P of (**c**) 0.5 V or (**d**) -0.5 V. (**e**) A2/A1 index of Δ*mtrC* strain carrying *mtrC* Buffer gate (+*mtrC*) or empty vector plasmid (+empty) under steady-state protein expression. A2/A1 index *p* values for pairs indicated from top to bottom $p = 0.0046$, $p = 0.0045$, and $p = 0.0149$. (**f**) Channel current I_{DS} responding to the continuous voltage pulses with V_P of 0.5 V or -0.5 V. Faded lines represent the raw I_{DS}, while the bolded lines represent I_{DS} baselines after filtering out the pulses. (**g, h**) One-phase exponential fitting of the I_{DS} baselines for each continuous 4-pulse session, with V_P equal to (**g**) 0.5 V or (**h**) -0.5 V. (**i**) The corresponding time constants of the fitting results. Time constant *p* values for the indicated pair $p = 1.0 \times 10^{-5}$. In panel (a) to (e), data show the mean ± SD of 3 biological replicates. In panel (e) and (i), unpaired two-tailed Student's t-tests were performed without adjustments for multiple comparisons, n.s. represents $p > 0.05$.

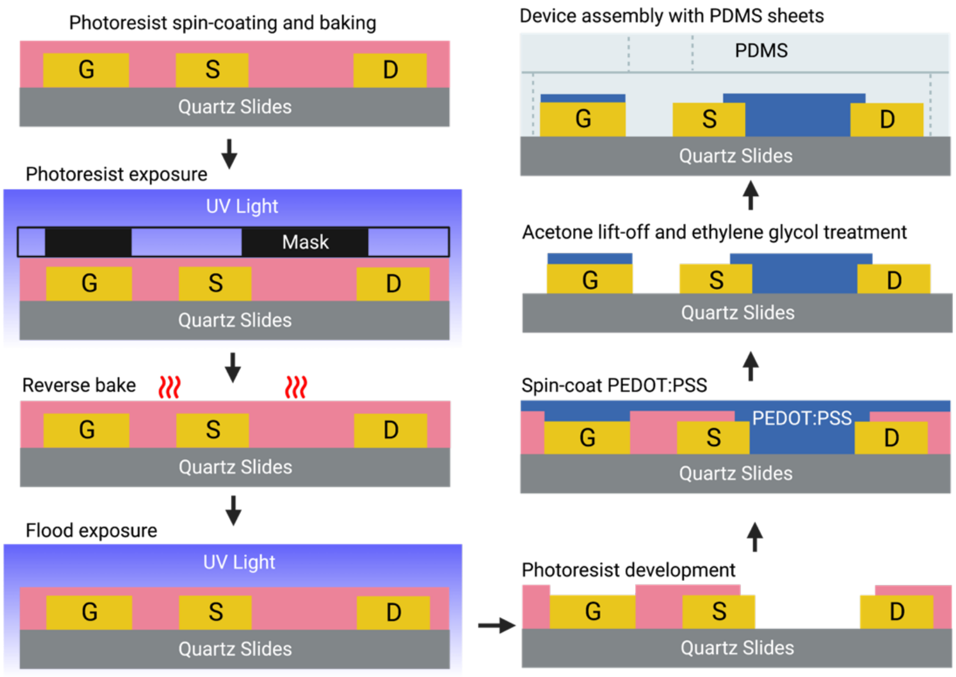

Figure S6. Cartoon illustration of the major OECT channel fabrication and assembly steps. Created with BioRender.com.

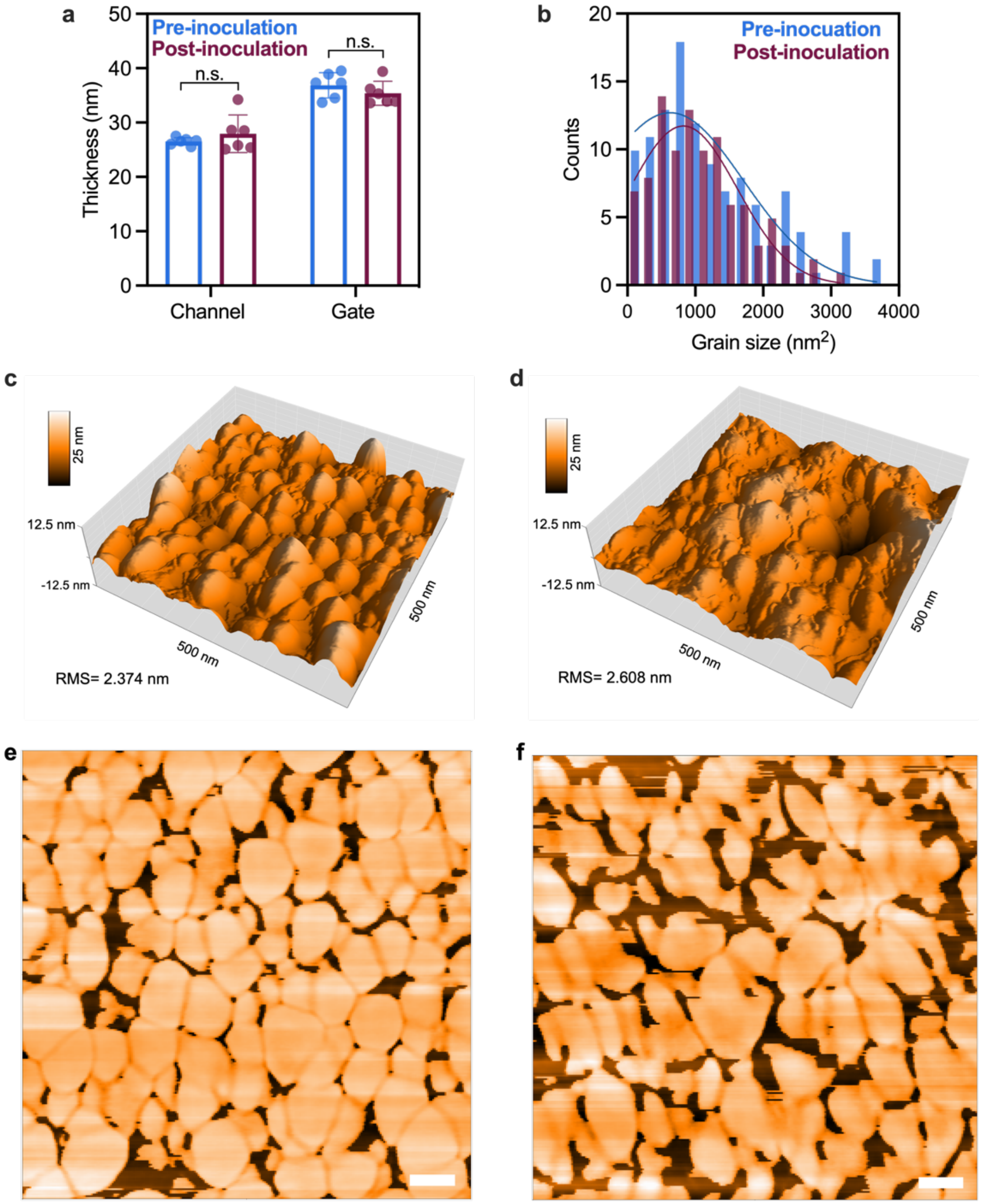

Figure S7. Morphological characterization of PEDOT:PSS channel and gate-tip coating. (**a**) Thickness of PEDOT:PSS films derived from AFM scans. OECTs were inoculated with *S. oneidensis* and operated with constant bias voltages at the gate V_{GS} = 0.2 V and drain V_{DS} = -0.05 V for 48 hours. (**b**) Histogram and Gaussian fit (lines) of PEDOT grain size in the channel region based on (e) and (f). Representative surface topologies of the PEDOT:PSS channel (**c**) prior to and (**d**) after the S. oneidensis incubation. Representative phase images of the PEDOT:PSS channel (**e**) prior to and (**f**) after the incubation, scale bars represent 50 nm. AFM measurements were performed on 6 independent samples. Panel (c) to (f) presents a zoomed-in morphology graph extracted from 2 representative samples. In panel (a), unpaired two-tailed Student's t-tests were performed without adjustments for multiple comparisons, n.s. represents $p > 0.05$.

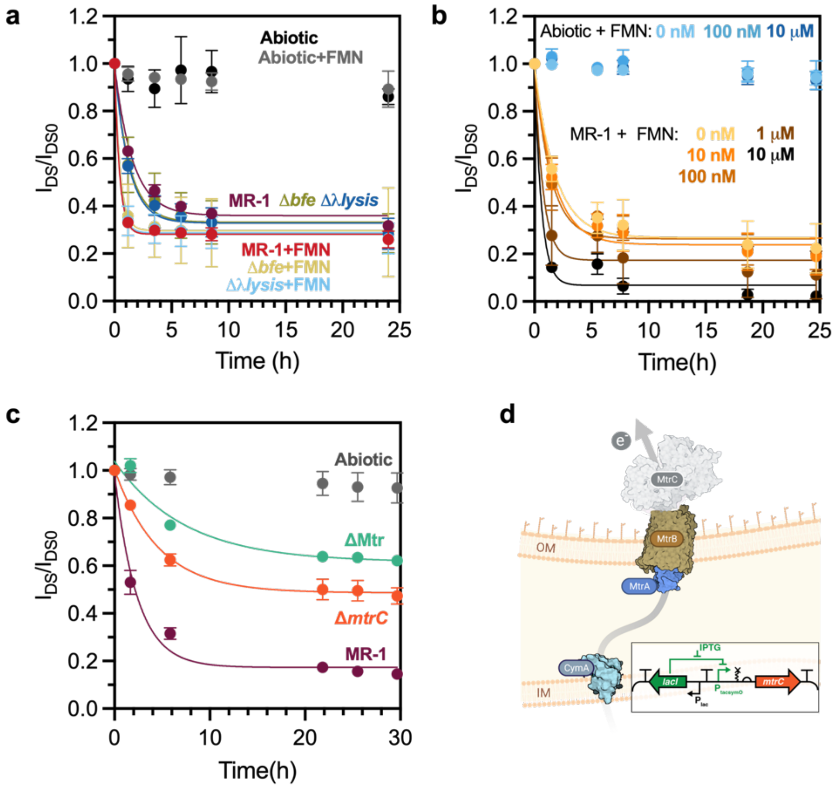

Figure S8. OECT response to different extracellular electron transfer (EET) mechanisms. (**a**) The I_{DS}/I_{DS0} curves of knockout strains with and without the addition of exogenous flavin mononucleotide (FMN) (1μM), and (**b**) *S. oneidensis* MR-1 cells with varying exogenous FMN concentrations. Initial inocula were adjusted to OD_{600} of 0.05. (**c**) The I_{DS}/I_{DS0} curves of Δ*mtrC*, ΔMtr, and MR-1 strains with initial inoculation OD_{600} at 0.1. (**d**) Cartoon illustration of the Δ*mtrC* strain and (d, insert) diagram of the *mtrC* Buffer gate controlled by the IPTG inducer. Faded shapes indicate removed proteins with genomic deletion and inhibition of electron transfer through the inner (IM) and outer (OM) membranes. Data show the mean ± SD of 3 biological replicates. Figure (d) created with BioRender.com.

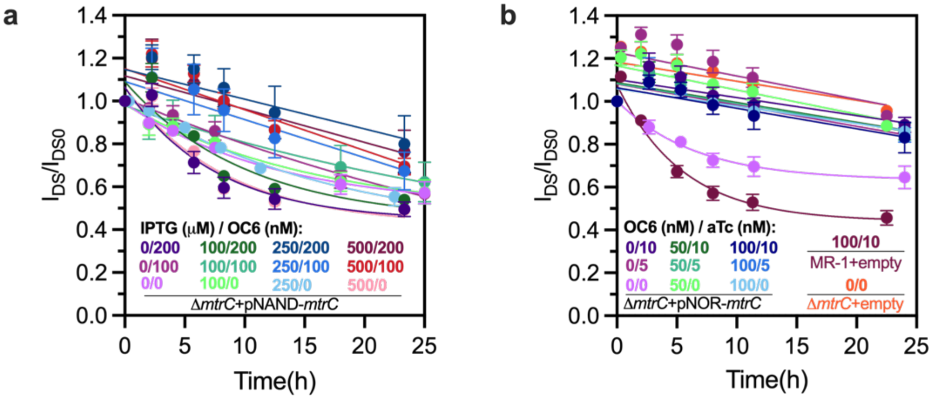

Figure S9. Channel current I_{DS}/I_{DS0} curves of strains carrying the Boolean logic gates plasmids. (a) ∆*mtrC* strain carrying NAND Boolean *mtrC* plasmids. (b) ∆*mtrC* strain carrying NOR Boolean *mtrC* plasmids. MR-1 strain and ∆*mtrC* strain carrying empty vectors were used as positive and negative controls, respectively. Inducible mutants were brought to steady-state expression under various inducer combinations and concentrations before inoculation. Data show the mean ± SD of 3 biological replicates.

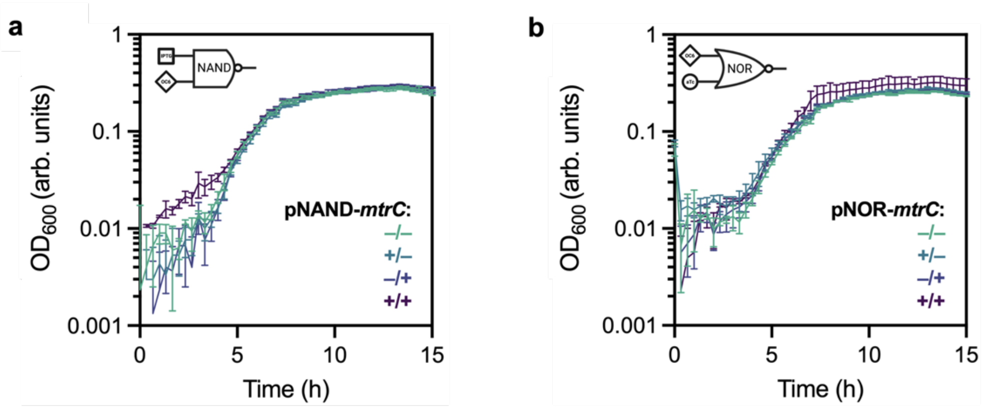

Figure S10. Growth curves of mutant strains carrying the Boolean logic gates plasmids. (**a**) NAND or (**b**) NOR Boolean *mtrC* plasmids under different inducer (IPTG, OC6, and aTc) combinations. Data show the mean ± SD of 3 biological replicates. Created with BioRender.com

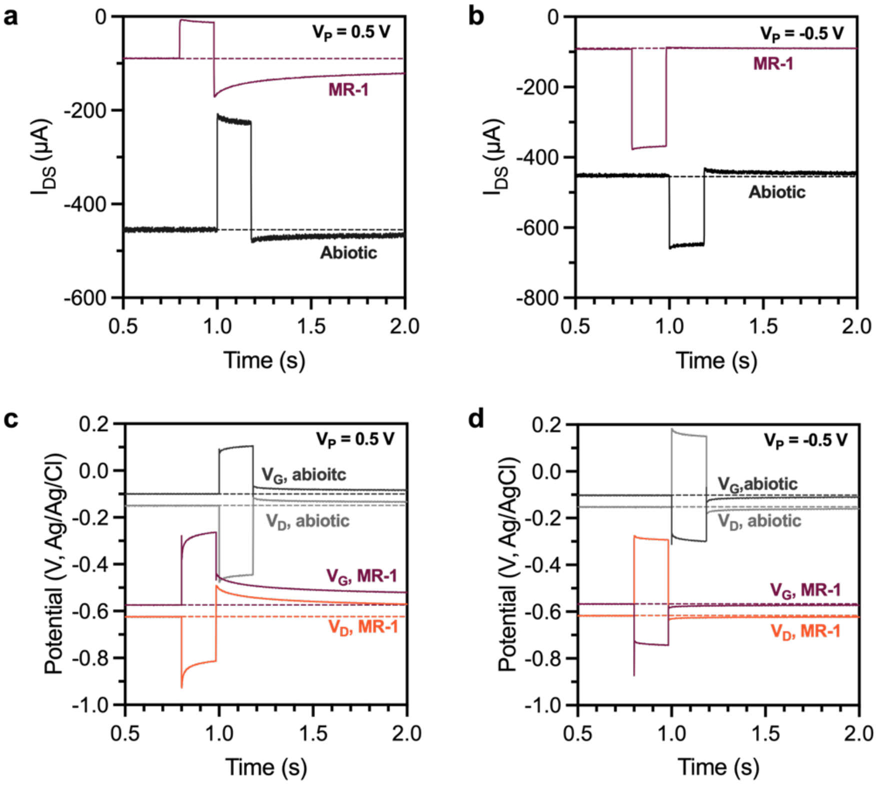

Figure S11. Measured channel currents I_{DS} and electrode potentials responding to gate pulses. Single gate pulses with pulse duration t_p = 1.5 s, pulse voltage V_P equal to (**a, c**) 0.5 V and (**b, d**) -0.5 V were applied between gate and source electrodes while constant drain voltage V_{DS} = -0.05 V was applied. Gate potential V_G and drain potential V_D were measured against Ag/AgCl pseudo-reference electrodes.

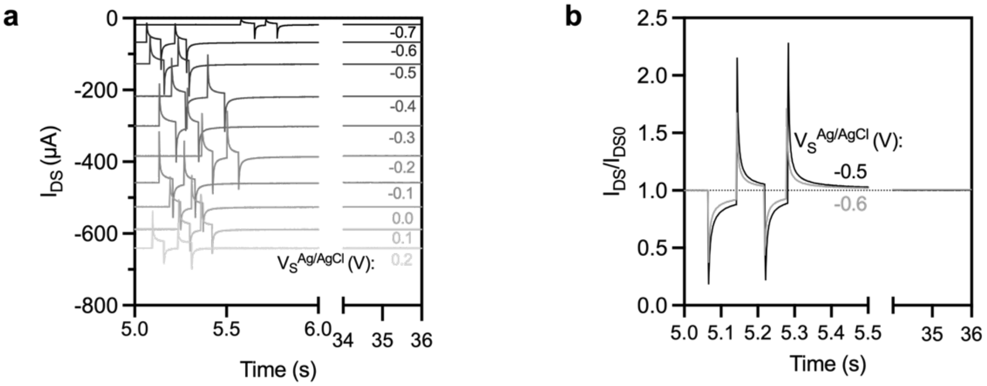

Figure S12. Paired-pulse responses of abiotic OECTs at varying electrochemical doping states. The source electrode potential (V_S) was constantly biased at the specified voltages against the Ag/AgCl pseudo-reference electrode. (**a**) Channel current I_{DS} of OECT with V_S biased from 0.2 V to -0.7 V in increment of 0.1 V. (**b**) Normalized I_{DS} for V_S biased at -0.5 V and -0.6 V.

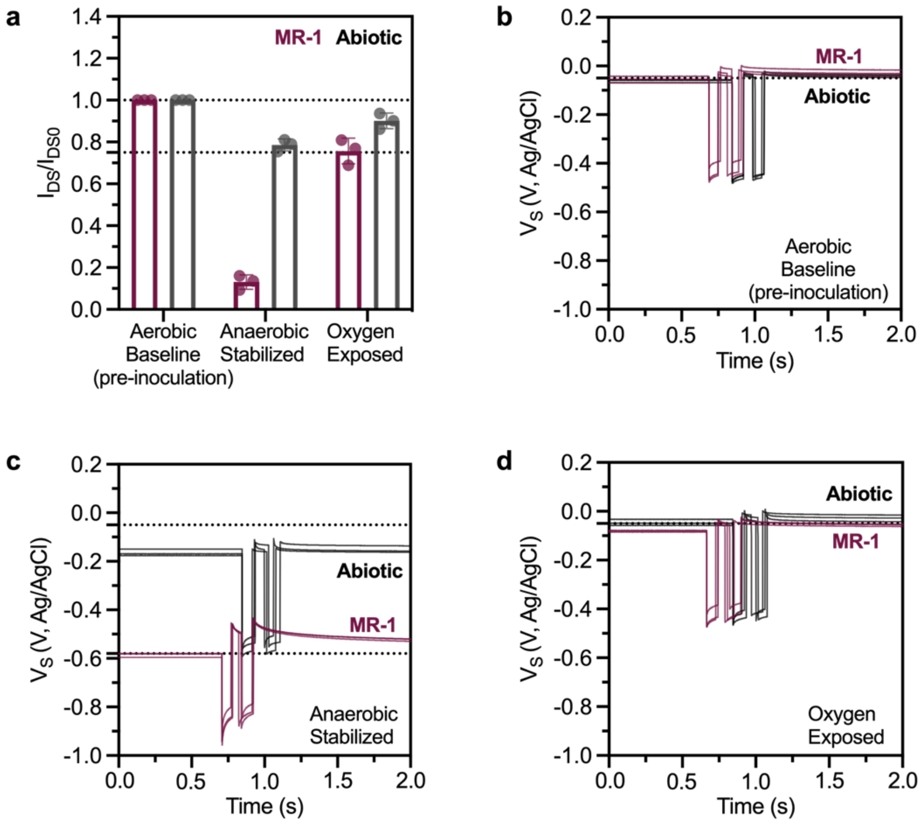

Figure S13. Electrochemical response of the hybrid OECTs under varying oxygen conditions. (**a**) Channel current I_{DS} were normalized to the pre-inoculation aerobic baseline values, with gate voltage V_{GS} = 0 V and drain voltage V_{DS} = -0.05 V. Paired pulse responses of pulse voltage V_P = 0.5 V and drain voltage V_{DS} = -0.05V were shown with the measured source electrode potentials against Ag/AgCl pseudo-reference electrode during (**b**) aerobic pre-inoculation condition, (**c**) anaerobic stabilization post-inoculation, and (**d**) subsequent re-oxygenation to ambient conditions. Data in panel (a) show the mean ± SD of 3 biological replicates.

References

1 Yoo, B., Dodabalapur, A., Lee, D. C., Hanrath, T. & Korgel, B. A. Germanium nanowire transistors with ethylene glycol treated poly(3,4-ethylenedioxythiophene): poly(styrene sulfonate) contacts. *Applied Physics Letters* **90** (2007). https://doi.org/Artn 072106 10.1063/1.2535710

2 Muller, J., Shukla, S., Jost, K. A. & Spormann, A. M. The mxd operon in Shewanella oneidensis MR-1 is induced in response to starvation and regulated by ArcS/ArcA and BarA/UvrY. *Bmc Microbiology* **13** (2013). https://doi.org/Artn 119 10.1186/1471-2180-13-119

3 Coursolle, D. & Gralnick, J. A. Modularity of the Mtr respiratory pathway of Shewanella oneidensis strain MR-1. *Molecular Microbiology* **77**, 995-1008 (2010). https://doi.org/10.1111/j.1365-2958.2010.07266.x

4 Coursolle, D. & Gralnick, J. A. Reconstruction of extracellular respiratory pathways for iron(III) reduction in Shewanella oneidensis strain MR-1. *Frontiers in Microbiology* **3** (2012). https://doi.org/ARTN 56 10.3389/fmicb.2012.00056

5 Kotloski, N. J. & Gralnick, J. A. Flavin Electron Shuttles Dominate Extracellular Electron Transfer by Shewanella oneidensis. *Mbio* **4** (2013). https://doi.org/ARTN e00553-12 10.1128/mBio.00553-12

6 Binnenkade, L., Teichmann, L. & Thormann, K. M. Iron Triggers lambda So Prophage Induction and Release of Extracellular DNA in Shewanella oneidensis MR-1 Biofilms. *Applied and Environmental Microbiology* **80**, 5304-5316 (2014). https://doi.org/10.1128/Aem.01480-14

7 Dundas, C. M., Walker, D. J. F. & Keitz, B. K. Tuning Extracellular Electron Transfer by Shewanella oneidensis Using Transcriptional Logic Gates. *Acs Synthetic Biology* **9**, 2301-2315 (2020). https://doi.org/10.1021/acssynbio.9b00517

8 Graham, A. J. *et al.* Transcriptional Regulation of Synthetic Polymer Networks. *bioRxiv*, 2021.2010.2017.464678 (2021). https://doi.org/10.1101/2021.10.17.464678
